# Assistance Robotics and Biosensors

**DOI:** 10.3390/s18103502

**Published:** 2018-10-17

**Authors:** Fernando Torres, Santiago T. Puente, Andrés Úbeda

**Affiliations:** 1 Department of Physics, System Engineering and Signal Theory, University of Alicante, 03690 Alicante, Spain; santiago.puente@ua.es (S.T.P.); andres.ubeda@ua.es (A.Ú.); 2 Computer Science Research Institute, University of Alicante, 03690 Alicante, Spain

**Keywords:** electromyographic (EMG) sensors, electroencephalographic (EEG) sensors, assistance robotics applications, robotic exoskeletons, robotic prostheses, advanced biomedical signal processing

## Abstract

This Special Issue is focused on breakthrough developments in the field of biosensors and current scientific progress in biomedical signal processing. The papers address innovative solutions in assistance robotics based on bioelectrical signals, including: Affordable biosensor technology, affordable assistive-robotics devices, new techniques in myoelectric control and advances in brain–machine interfacing.

## 1. Introduction

In recent years, the use of bioelectrical information to enhance traditional motor-disability assistance has experienced significant growth, mostly based on the development and improvement of biosensor technology and the increasing interest in solving accessibility limitations in a more natural and effective way. For that purpose, control outputs are directly decoded from the user’s biological information. Biomedical signals, recorded from cortical or muscular activity, are used to interact with external devices, such as robotics exoskeletons or assistive robotic arms or hands. However, efforts are still needed to make these technologies affordable for end users, as current biomedical devices are still mostly present in rehabilitation centers, hospitals and research facilities.

## 2. Contributions

This Special Issue collected ten outstanding papers covering different aspects of assistance robotics and biosensors. In the following, a brief summary of the scope and main contributions of each of these papers is provided as a teaser for the interested reader.

One of the most important issues in assistive robotics is helping people with special needs or disabilities to adequately perform rehabilitation exercises in the friendliest way. In “A High-Level Control Algorithm Based on sEMG Signalling for an Elbow Joint SMA Exoskeleton” [1] the authors designed a high-level control algorithm capable of generating position and torque references from surface electromyography signals (sEMG). They applied this algorithm to a shape memory alloy (SMA)-actuated exoskeleton used in active rehabilitation therapies for elbow joints.

In the same field of assistance, the paper “Intelligent Multimodal Framework for Human Assistive Robotics Based on Computer Vision Algorithms” [2] shows a multimodal interface based on computer vision, which has been integrated into a robotic system together with other sensory systems (electrooculography (EOG) and electroencephalography (EEG)). The results were part of an European project, AIDE, whose purpose is to contribute to the improvement of current assistance technologies.

Undoubtedly, rehabilitation tasks require friendly systems and exoskeletons that are at the same time more precise. In this sense, the improvement of hardware systems is crucial. In the paper “ED-FNN: A New Deep Learning Algorithm to Detect Percentage of the Gait Cycle for Powered Prostheses” [3] the authors propose a novel gait detection algorithm that can predict a full gait cycle discretized within a 1% interval. In addition, the system provides an opportunity to eliminate detection delays for real-time applications.

In the case of assistive robotics, another field of great interest is creating equipment and friendly environments for people with physical movement disabilities. In this context, the authors of the paper “A Vision-Driven Collaborative Robotic Grasping System Tele-Operated by Surface Electromyography” [4] propose an interface that combines computer vision with electromyography, aiming to allow a person with impeded movement to teleoperate a robotic hand. Experiments were carried out on basic operations of the grasping and shifting of objects.

The problem of more reliable myoelectric systems is addressed in the paper “Virtual Sensor of Surface Electromyography in a New Extensive Fault-Tolerant Classification System” [5]. The authors propose extending the use of virtual sensors used in other research fields to the myoelectric field. With this, they provide a new, extensive, fault-tolerant classification system to maintain the classification accuracy after the occurrence of the following contaminants: ECG interference, electrode displacement, movement artifacts, power line interference, and saturation. The time-varying autoregressive moving average (TVARMA) and time-varying Kalman filter (TVK) models were compared to define the most robust model for the virtual sensor.

In “Effects of tDCS on Real-Time BCI Detection of Pedaling Motor Imagery” [6] the authors sought to strengthen the cortical excitability over the primary motor cortex (M1) and the cerebro-cerebellar pathway by means of a new transcranial direct current stimulation (tDCS) configuration to detect lower limb motor imagery (MI) in real time using two different cognitive neural states: relaxed and pedaling MI. In this case, the use of software or hardware techniques with the purpose of improving the reception of signals was again treated.

The use of assistive robotics is justified when it improves the life of people with certain disabilities. In this sense, the use of appropriate signals for each case is of great importance. In “A Novel Feature Optimization for Wearable Human–Computer Interfaces Using Surface Electromyography Sensors” [7], the authors carried out a study of the signals and selection of optimal-feature selection made according to a modified entropy criteria (EC) and Fisher discrimination (FD) criteria. The feature selection results were evaluated using four different classifiers, and compared with other conventional feature subsets. These experiments validated the feasibility of the proposed real-time wearable HCI system and algorithms, providing a potential assistive device interface for persons with disabilities.

In the same field of achieving improvements for people with certain disabilities, the paper titled “Evaluating the Influence of Chromatic and Luminance Stimuli on SSVEPs from Behind-the-Ears and Occipital Areas” [8] presents a study of chromatic and luminance stimuli in low-, medium-, and high-frequency stimulation to evoke steady-state visual evoked potential (SSVEP) in the behind-the-ears area. These findings will aid in the development of more comfortable, accurate and stable BCI with electrodes positioned in the behind-the-ears (hairless) areas.

The use of exoskeletons in rehabilitation therapies is increasingly widespread and it is one of the most promising and expected future lines in the field of assistive robotics. The authors of “Disturbance-Estimated Adaptive Backstepping Sliding Mode Control of a Pneumatic Muscle-Driven Ankle Rehabilitation Robot” [9] propose the improvement of a therapeutic robot for the rehabilitation of ankle injuries. To do this, they proposed a new method of adaptive backstepping sliding mode control (ABS-SMC) in order to solve the PM’s nonlinear characteristics during operation and to tackle the human–robot uncertainties in rehabilitation.

The correct adaptation of an exoskeleton to patients with knee problems is dealt with in “Knee Impedance Modulation to Control an Active Orthosis Using Insole Sensors” [10]. In this case, the authors propose a method for online knee impedance modulation that generates variable gains through the gait cycle according to the users’ anthropometric data and gait sub-phases recognized through footswitch signals.
